# Equity issues in gender-affirming medical care in Kerala: a reflective commentary

**DOI:** 10.1186/s12939-023-01981-9

**Published:** 2023-09-20

**Authors:** K. Rajasekharan Nayar, S. Vinu

**Affiliations:** Global Institute of Public Health Ananthapuri Hospitals and Research Institute Thiruvananthapuram, Thiruvananthapuram, Kerala India

## Abstract

Gender-affirming medical care is the provision of transition-related medical services that support a transgender person’s own gender identity. Gender transitioning is a process that requires not only social support but also psychological and medical support, This paper attempts to document the challenges faced by transgender individuals (TG) especially in the context of gender affirming medical care in the Kerala context. The transition process is extremely complex as the preference for such process is varied. Some transgender individuals preferred social transition and/or medical transition to align their gender expression with their gender identity, while others chose to have a gender expression or identity outside the traditional gender binary. In Kerala, despite proactive policy and positive legal support, transgender individuals face many challenges in gender-affirming medical care which include lack of family support and equity-related issues with respect to a number of social support institutions including health services. A few possible interventions are suggested such as changes in medical curriculum, more active State support and sensitization of the society including health workers.

Transgender individuals experience lifelong dysphoria due to incompatibility in gender assigned at birth with their gender identity. Many such individuals attempt gender transitioning which is a process that requires not only social support but also psychological and medical support. This commentary attempts to trace the challenges faced by transgender individuals (TG) especially in the context of gender affirming medical care in the Kerala. For this purpose, we did a review of the limited literature available using pubmed, google scholar etc. in order to contextualise the problems that the TG community face while undergoing transition treatments. We also used insights derived from our own qualitative work among the TG community in Kerala. The existing policy framework was also useful in writing this commentary.

## Quality of life of transgender population

The health and health services utilization dynamics of TG individuals are influenced by their overall Quality of Life (QoL). It is a known fact that such individuals are stigmatized in our society and are being discriminated in every aspect of life. These might have serious implications on their quality of life (QoL). There are only a few studies which focus on their overall quality of life. A study conducted among 79 adult transgender people residing in Burdwan district, West Bengal found positive direction with respect to quality of life (i.e. higher scores denoting higher quality of life). There is statistically significant association between marital status, current living status and main source of income with overall QoL score. Monthly income and education of the study population had a significant positive correlation with their QOL score while a significant negative correlation was found between age and QoL score [1].

A comparison between 37 gender incongruent individuals from hijra community (hijra cohort) and 37 apparently healthy employees (control cohort) of a tertiary care hospital at Kolkata showed that both ‘hijra’ cohort and control cohort, rated their QoL equal in the eight dimensions of general health perceptions, physical function, role limitations due to physical health problems, pain, general mental health, social functioning, and vitality [2]. In Kerala, similar to the general population, income and other social factors play an important role in maintaining QoL of transgender population [3]. However, there are many specificities especially with respect to everyday experiences which have implications for health and which result in health inequities for TG people.

## Transgender policy landscape

There may be many challenges which transgender individuals face in Kerala despite introducing transgender friendly laws and it is important to inform and improve social and health policies and programs in the state based on these challenges. Following the directives from Supreme Court judgment of 2014, Kerala became the first Indian state to implement a comprehensive policy to enforce the constitutional rights of transgender people [4]. Government of India also brought in several initiatives such as Transgender Act in 2019 and following it, the Transgender Rules in 2020 [5]. Both recognize the intense discrimination and violence faced by TG persons in the society and prescribe appropriate legal and administrative actions.

In Kerala, some initiatives have been undertaken by a few State organizations after the state policy and central acts and rules. The state police through a circular in 2022 instructed its members to treat the TG persons with dignity and respect their rights in society [6]. The University of Kerala also developed a special protection scheme in 2021 in order to remove impediments against TG persons from pursuing higher education and to protect their identity and self-respect of such students [7].

## Health challenges among the Transgender

Health challenges for transgender people (TG) are multifactorial with risks including systematic, social, and economic marginalization, pathologization, stigma, discrimination, and violence [8]. They also face such trends in healthcare systems and settings. It was found that many transgender individuals resorted to postponing needed medical care when sick or injured and they also postponed routine preventive care [9]. This postponement many times led to medical emergencies that required special emergency or urgent care [10]. The treatment-seeking behavior also shows a lack of trust in doctors, leading to seeking treatment and care only in case of aggravated conditions, which inflates the cost of treatment and the burden of disease [11], [12]. High rates of various negative health outcomes and related psychosocial risk factors were significantly elevated in TGD (Transgender and Gender Diverse) AMAB (assigned male at birth) youth, including transgender women and non-binary AMAB youth compared to their cisgender sexual minority peers indicating that these youth maybe at even higher risk for adverse health outcomes across multiple domains [13].

Transgender individuals have a variety of health needs that require accessible, affordable, and quality healthcare. For those seeking medical transition, this can include gender-affirming medical care such as hormone therapy, surgery, and support services like counselling. Insurance-based denials are common barriers for transgender and non-binary individuals in accessing medically necessary gender-affirming care [14]. Their experiences with healthcare discrimination are pervasive and include providers refusing to offer gender-affirming medical care, asking unnecessary questions about gender, unrelated to the purpose of the healthcare visit, and lacking knowledge of trans-related health issues. Evidently, regarding transition treatments, some of these challenges could be severe than normal health-seeking experiences and need to be documented for evolving and ensuring an equity-based health program.

## The dynamics of transition

Gender-affirming medical care is the provision of transition-related medical services that support a transgender person’s own gender identity [15]. Some transgender individuals preferred social transition (e.g., change their name, pronoun, gender expression) and/or medical transition (e.g., cross-sex hormones, surgery) to align their gender expression with their gender identity, while others preferred to have a gender expression or identity outside the traditional gender binary (e.g. gender nonconforming people) [8]. Transitioning is not a universal process among them and even those who opt for it may not follow all the needed steps. Those who carry out the transition process according to their own will have a safer self-image and have lower depression rates and better communication in their relationships [16]. This means that gender transitioning is a process that requires not only social support but psychological and medical support as well [17].

The Transgender Survey, Kerala (2014) showed that due to societal and family pressures, 70–80% of transgender people enter into married life and have children, but most of them part ways within a month or a year [18]. The survey also reported that 54% of transgender people in Kerala earn < 5000 as monthly income and only 11.6% have regular jobs. About 90% of transgender people in Kerala drop out of school due to taunting from fellow students, teachers. The survey estimated the presence of more than 25,000 transgender people and among them overwhelmingly 32% attempted suicide at least once in their life.

The survey estimated that although 52% felt a need to change their physical appearance through medical or surgical interventions, only 9% could do so. Social Justice Department of Kerala has implemented several provision that can be accessed by anyone with the ID card issued by them such as Sex Reassignment Surgery treatment cost reimbursement, availing stay facility at shelter homes, and launch of 24 × 7 transgender helpline number to provide distress counselling [10]. Facilities for gender-affirmative health care in government hospitals are still lacking. In the past, transgender individuals used to go to neighbouring states and undergo surgeries and some end up with lifelong complications [10]. From the survey and the available literature, it can be surmised that the gender transitioning process is a difficult phase due to lack of family support and equity-related issues with respect to a number of social support institutions including health services. This is despite a proactive policy framework in the state.

## Concluding observations: some possible interventions

A few key interventions need to be carried out in order to ensure an equity-oriented transitioning process which may help in overcoming some of the challenges identified in this commentary. Gender transitioning is a complex process and is closely linked to their socio-cultural positioning in the society. Especially, medical personnel can play a very positive role in this ‘life-changing process’ which is not just a surgical procedure but has social, economic and cultural components.

### Changes in Medical Curriculum

Transgender health has yet to gain widespread curricular exposure but efforts towards incorporating transgender health into both undergraduate and graduate medical education curriculum are nascent. There is no consensus on the exact educational interventions that should be used to address transgender health. There should be efforts to create a curriculum which can help in empowering physicians to identify and change the systemic barriers to care that cause transgender health inequities as well as improve knowledge about transgender-specific care. Barriers to increased transgender health exposure includes limited curricular time, lack of topic-specific competency among faculty, and underwhelming institutional support [19].

### More active state support

There is a need to evolve clear guidelines regarding the nature of State support for the transitioning process which should also state the timeframe for such support. The state at present supports by reimbursing the treatment costs. However, the treatments days often result in unemployment and the typical bureaucratic delays in reimbursement lead to more suffering.

### Sensitization of the society

There is a need to sensitize the society as well as health workers regarding the challenges faced by transgender individuals to lead a normalised existence in our society. Apart from trained medical professionals, transgender counselling centres and other facilities, their freedom to use public toilets and transportation should also be addressed. Acceptance of the notion of third gender or transgender is a slow process and there is a long way to go in achieving it in Kerala.

## Data Availability

Not applicable.
